# Predicting Diabetes and Estimating Its Economic Burden in China Using Autoregressive Integrated Moving Average Model

**DOI:** 10.3389/ijph.2021.1604449

**Published:** 2022-01-20

**Authors:** Di Zhu, Dongnan Zhou, Nana Li, Bing Han

**Affiliations:** Department of Biostatistics, School of Public Health, China Medical University, Shenyang, China

**Keywords:** China, diabetes, ARIMA model, economic burden, prevalence trend

## Abstract

**Objectives:** To predict the number of people with diabetes and estimate the economic burden in China.

**Methods:** Data from natural logarithmic transformation of the number of people with diabetes in China from 2000 to 2018 were selected to fit the autoregressive integrated moving average (ARIMA) model, and 2019 data were used to test it. The bottom-up and human capital approaches were chosen to estimate the direct and indirect economic burden of diabetes respectively.

**Results:** The number of people with diabetes in China would increase in the future. The ARIMA model fitted and predicted well. The number of people with diabetes from 2020 to 2025 would be about 94, 96, 97, 98, 99 and 100 m respectively. The economic burden of diabetes from 2019 to 2025 would be about $156b, $160b, $163b, $165b, $167b, $169b and $170b respectively.

**Conclusion:** The situation of diabetes in China is serious. The ARIMA model can be used to predict the number of people with diabetes. We should allocate health resources in a rational manner to improve the prevention and control of diabetes.

## Introduction

Diabetes is a chronic metabolic disease in which the body cannot use or produce enough insulin efficiently [1], mainly related to genetic and environmental factors. People with diabetes need to keep appropriate blood sugar levels through medication and diet, etc. Failure to control blood glucose effectively for a long time leads to various acute and chronic complications which eventually lead to disability or death, such as diabetic ketoacidosis, diabetic nephropathy, diabetic foot and so forth. Diabetes is one of the significant causes of death worldwide [2] and it imposes a substantial global burden [3]. From 2000 to 2019, the number of people with diabetes increased from 218 million to 460 million worldwide [4]. During the same time period, the number of people with diabetes increased from 48 million to 92 million in China, which made China the country with the highest number of people with diabetes in the world in 2019 [4]. Besides, it was estimated that the number of undiagnosed diabetes patients was about 65 million in China in 2019 [5]. Therefore, diabetes becomes an extremely serious public health challenge in the world and in China.

In 2019, the number of people aged 60 and over in China was approximately 254 million, which accounted for about 18.1% of the total population [6]. Since the prevalence of diabetes increases with age [7], such a large susceptible population inevitably leads to the increase in the number of people with diabetes. Therefore, it is particularly important to forecast the prevalence trends of diabetes in China.

ARIMA model is one of the analysis methods for time series. This model is based on an adjusted version of the observed values and its objective is to reduce the difference between the values generated in the model and the observed values to as close to zero as possible [8]. This model is widely used in the medical field. For example, with data on prostate cancer in Australia from 1982 to 2013, ARIMA model was used to predict the incidence of prostate cancer from 2014 to 2022 [9]. The ARIMA model was used to predict Alzheimer’s disease mortality in the United States from 2019 to 2023 [10]. In addition, ARIMA model has a wide range of applications in fields such as meteorology [11], economics [12] and agriculture [13].

The high prevalence of diabetes not only affects the health condition of people with diabetes seriously, but also causes a heavy economic burden of the disease on patients, their families, and society [14]. Economic burden of disease refers to the economic loss due to disease and its complications, including direct economic burden, indirect economic burden and intangible economic burden [15]. The direct economic burden of diabetes refers to the costs incurred during the treatment and rehabilitation of diabetes, mainly including outpatient costs, inpatient costs, and drug costs [16]. Indirect economic burden refers to the loss of productivity due to morbidity and premature mortality caused by diabetes [17]. According to the estimation of International Diabetes Federation, the global health expenditure of diabetes was approximately $760 billion in 2019, and the countries with the heaviest health expenditure of diabetes were: the United States at $295 billion, China at $109 billion, and Brazil at $52 billion [5]. Previous studies showed that the indirect economic burden of diabetes cost between 30 and 40% of the total economic burden in 2015 [3]. The economic burden of diabetes is increasing rapidly. If no effective measures are taken, diabetes will become one of the major challenging factors of the economic and social development.

In this study, the ARIMA model is used to fit the log-transformed annual number of people with diabetes in China from 2000 to 2018, select the number of people with diabetes in 2019 for model testing, and predict the future number of people with diabetes in China. The direct economic burden of diabetes and the indirect economic burden of diabetes are estimated using the bottom-up approach and the human capital approach respectively. The purpose of the research is to provide data support for diabetes prevention and control in China, and to establish a theoretical basis for relevant national departments to improve the diabetes-related health policy and rationalize the allocation of health resources.

## Methods

### Data

Data on the number of people with diabetes from 2000 to 2019 for each year and disability-adjusted life years (DALYs) were obtained from the Global Burden of Disease (GBD) (http://ghdx.healthdata.org/gbd-results-tool) (Supplemental Table S1). The Chinese gross national income (GNI) per capita in 2019 was obtained from the National Bureau of Statistics of China (http://www.stats.gov.cn/tjsj/sjjd/202008/t20200807_1781473.html). The conversion between USD and RMB was based on the exchange rate in 2019 published by National Bureau of Statistics of China. Data were analyzed using R4.1.0.

## ARIMA Model

### Model Introduction

The ARIMA (*p*, *d*, *q*) model (*p* is the autoregressive term which express the relationship between current and historical values, *d* is the number of differences, and *q* is the moving average term which is used to eliminate random fluctuations) in which AR stands for autoregressive, MA stands for moving average, and I stands for integrated [18]. The p-order AR process is: $X_{t}=\phi_{0}+\phi_{1}X_{t-1}+\phi_{2}X_{t-2}+\ldots +\phi_{p}X_{t-p}+\varepsilon_{t}$ . The q-order MA process is: $X_{t}=\mu +\varepsilon_{t}-\theta_{1}\varepsilon_{t-1}-\theta_{2}\varepsilon_{t-2}-\ldots -\theta_{q}\varepsilon_{t-q}$ . The combination AR and MA generates the ARMA (*p*, *q*) model suitable for the stationary time series. Therefore, the operating step of the ARIMA (*p*, *d*, *q*) model is to select the ARMA (*p*, *q*) model after differencing the non-stationary series [19]. It is applicable to variables measured at equal or almost equal intervals of time [20]. The structure of ARIMA (*p*, *d*, *q*) model is as follows:

If $\left\{X_{t}\right\}$ is a non-stationary time series, it is a stationary ARIMA (*p*, *d*, *q*) process after differencing of $\left\{\nabla^{d}X_{t}\right\}$ , that is:

$$\{\begin{array}{c} \phi \left(B\right)\nabla^{d}X_{t}=\theta \left(B\right)\varepsilon_{t}\\ E\left(\varepsilon_{t}\right)=0,Var\left(\varepsilon_{t}\right)=\sigma_{\varepsilon }^{2},E\left(\varepsilon_{t}\varepsilon_{s}\right)=0,s\neq t\\ E\left(X_{s}\varepsilon_{t}\right)=0,\forall_{s}<t \end{array}$$ (1)

In this formula: $\nabla^{d}={\left(1-B\right)}^{d}$ ; $\phi \left(B\right)=1-\phi_{1}B-\ldots -\phi_{p}B^{p}$ is the autoregressive coefficient polynomial of the stationary reversible ARMA (*p*, *q*) model; $\theta \left(B\right)=1-\theta_{1}B-\ldots -\theta_{q}B^{q}$ is the moving average coefficient polynomial of the stationary reversible ARMA (*p*, *q*) model; $\varepsilon_{t}$ is the white noise series with zero mean; $\varepsilon_{1}$ is the predicted value minus the measured value, and *B* is the delay operator.

ARIMA (*p*, *d*, *q*) can also be represented by the following function:

$$X_{t}=\varepsilon_{t}+\Psi_{1}\varepsilon \left(t-1\right)+\Psi_{2}\varepsilon \left(t-2\right)+\ldots$$ (2)

$$X_{t}=\Psi \left(B\right)\varepsilon_{t}$$ (3)

### Modeling Process

We established a time series after natural logarithmic transformation of the number of people with diabetes in China from 2000 to 2018, and used ARIMA model to forecast the number of people with diabetes in China from 2019 to 2025. The modeling process was as follows: The first step was the unit root test. The ARIMA (*p*, *d*, *q*) model was applied to non-stationary series, so we checked whether the series was non-stationary. We chose the Augmented Dickey Fuller (ADF) test [11] to check if the series is stationary, and to determine the value of the model order *d*. The second step was the model order determination. Thus, we used the autocorrelation function (ACF) graph and partial autocorrelation function (PACF) graph to select the *p* and *q*. The third step was the model testing and diagnosis. The white noise test of residuals was performed using the *Box.test* function [11]. The Lagrange multiplier (LM) test of residuals was performed using the *ArchTest* function [21]. The model passed the above test (*p* > 0.05), indicating that the model was applicable to the time series of the number of people with diabetes. The final step was the prediction. We predicted the number of people with diabetes from 2019 to 2025. The model fitting effects were evaluated by the root mean squared error (RMSE), mean absolute error (MAE), mean absolute percentage error (MAPE), average relative error and coefficient of determination $\left(R^{2}\right)$ . The model prediction effects were evaluated by relative error. The model fitted and predicted values were transformed using the exp function to calculate the fitted and predicted values of the number of people with diabetes.

## Economic Burden of Diabetes

### Model Introduction

Bottom-up approach is a method to estimate the economic burden of disease by the product of unit cost and the number of patients [22]. The human capital approach is a method of calculating future income that is reduced due to mortality and/or morbidity [23].

### Total Economic Burden Estimation Process

Since the intangible economic burden was difficult to express in monetary terms, we chose the following method to estimate the total economic burden [24].

Total economic burden of diabates=direct economic burden of diabates+indirect economic burden of diabates (4)

### Direct Economic Burden Estimation Process

We chose the bottom-up approach to estimate the direct economic burden. The direct economic burden of diabetes from 2019 to 2025 was estimated by selecting the annual cost of treatment per capita of diabetes in 2019 published by the National Health Insurance Administration of China, the number of people with diabetes in 2019 published by GBD, and the number of people with diabetes from 2020 to 2025 predicted by using ARIMA model. The method was as follows:

Direct economic burden of diabetes=annual cost of treatment per capita of diabetes×the number of people with diabetes (5)

### Indirect Economic Burden Estimation Process

The human capital approach was used to estimate the indirect economic burden. The indirect economic burden of diabetes and indirect economic burden per capita in 2019 were estimated by selecting the GNI per capita in 2019, the DALYs of Chinese diabetes for different age groups in 2019 published by GBD, and the productivity weighted for different age groups: 0.15 for 0–14 years; 0.75 for 15–44 years; 0.80 for 45–59 years; and 0.10 for 60 years or older [25, 26]. To estimate the indirect economic burden of diabetes from 2019 to 2025, the methods were as follows:

Indirect economic burden of diabetes in 2019=GNI per capita in 2019×diabetes DALYs in 2019×productivity weights (6)

$$\begin{array}{l} \text{Indirect}\;\text{economic}\;\text{burden}\;\text{of}\;\text{diabetes}\;\text{in}\;2020\\ \left(2021,2022,2023,2024,2025\right)\\ =\text{indirect}\;\text{economic}\;\text{burden}\;\text{per}\;\text{capita}\;\text{in}\;2019\\ \times \text{the}\;\text{number}\;\text{of}\;\text{people}\;\text{with}\;\text{diabetes}\;\text{in}\;2020\\ \left(2021,2022,2023,2024,2025\right) \end{array}$$ (7)

## Results

### Prediction Results

**FIGURE 1:**
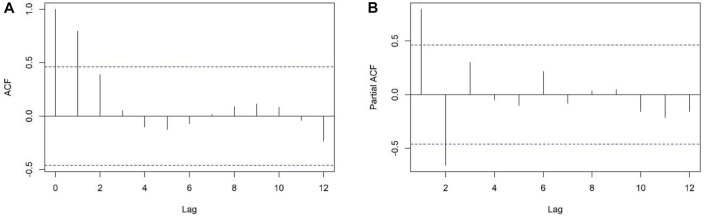
Autocorrelation function graph **(A)** and partial autocorrelation function graph **(B)** after differencing the time series, Predicting and Estimating Diabetes Burden, China, 2000–2018.

**FIGURE 2:**
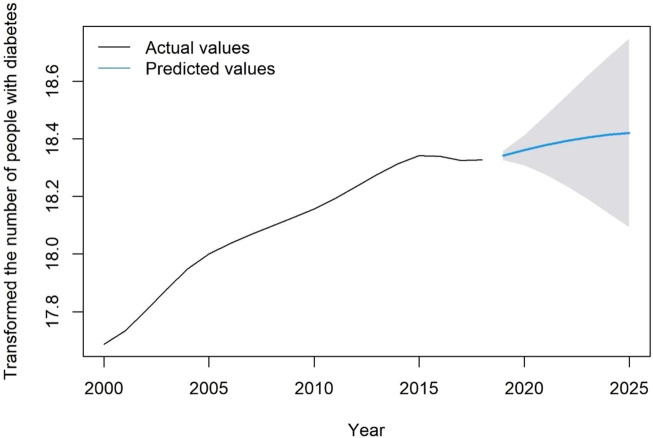
Fitted and predicted values for the number of people with diabetes after natural logarithmic transformation, Predicting and Estimating Diabetes Burden, China, 2000–2025.

We created a time series with the natural logarithmic transformation of the number of people with diabetes in China from 2000 to 2018. The ADF test was statistically significant (*p* < 0.05) after first differencing. We estimated the parameters p=2 and q=1 from ACF graph and PACF graph (Figure 1). The ARIMA (2,1,1) model passed the white noise test (*p* > 0.05), and the LM test (*p* > 0.05). AIC (-108.23), RMSE (0.0092), MAE (0.0069), MAPE (0.0379), average relative error (0.04%) and $R^{2}$ (0.99) indicated that the model fitted well. The relative error of prediction (0.03%) also indicated that the model predicted well. Therefore, we chose the ARIMA (2,1,1) model for prediction. The model predicted an increasing prevalence trend of diabetes in the future and the predicted value of diabetes prevalence in 2019 (92,462,626) was very close to the actual value (91,976,595). Therefore, the ARIMA (2,1,1) model can be used for the prediction of the number of people with diabetes in China (Figure 2), with the predicted values shown in Table 1.

**TABLE 1:** Prediction results of diabetes prevalence trend, Predicting and Estimating Diabetes Burden, China, 2000–2025.

Year	Actual values of the number of people with diabetes	Fitted/Predicted values of the number of people with diabetes
2000	47,990,633	47,149,316
2001	50,269,421	49,756,667
2002	53,901,499	53,010,040
2003	58,193,128	58,275,375
2004	62,428,480	62,341,707
2005	65,678,126	66,252,583
2006	68,103,597	67,694,049
2007	70,318,751	70,192,001
2008	72,414,074	72,283,590
2009	74,548,519	74,314,546
2010	76,781,094	76,632,405
2011	79,596,503	78,913,806
2012	82,982,973	82,884,375
2013	86,551,169	86,331,828
2014	89,875,882	89,977,447
2015	92,381,953	92,639,707
2016	92,191,000	94,041,868
2017	90,886,584	89,594607
2018	91,078,791	90,273,676
2019	**91,976,595**	**92,462,626**
2020	—	**94,182,961**
2021	—	**95,841,919**
2022	—	**97,276,002**
2023	—	**98,441,498**
2024	—	**99,351,897**
2025	—	**100,043,834**

The bold values in are the major results of the manuscript.

### Prevalence Trends

**FIGURE 3:**
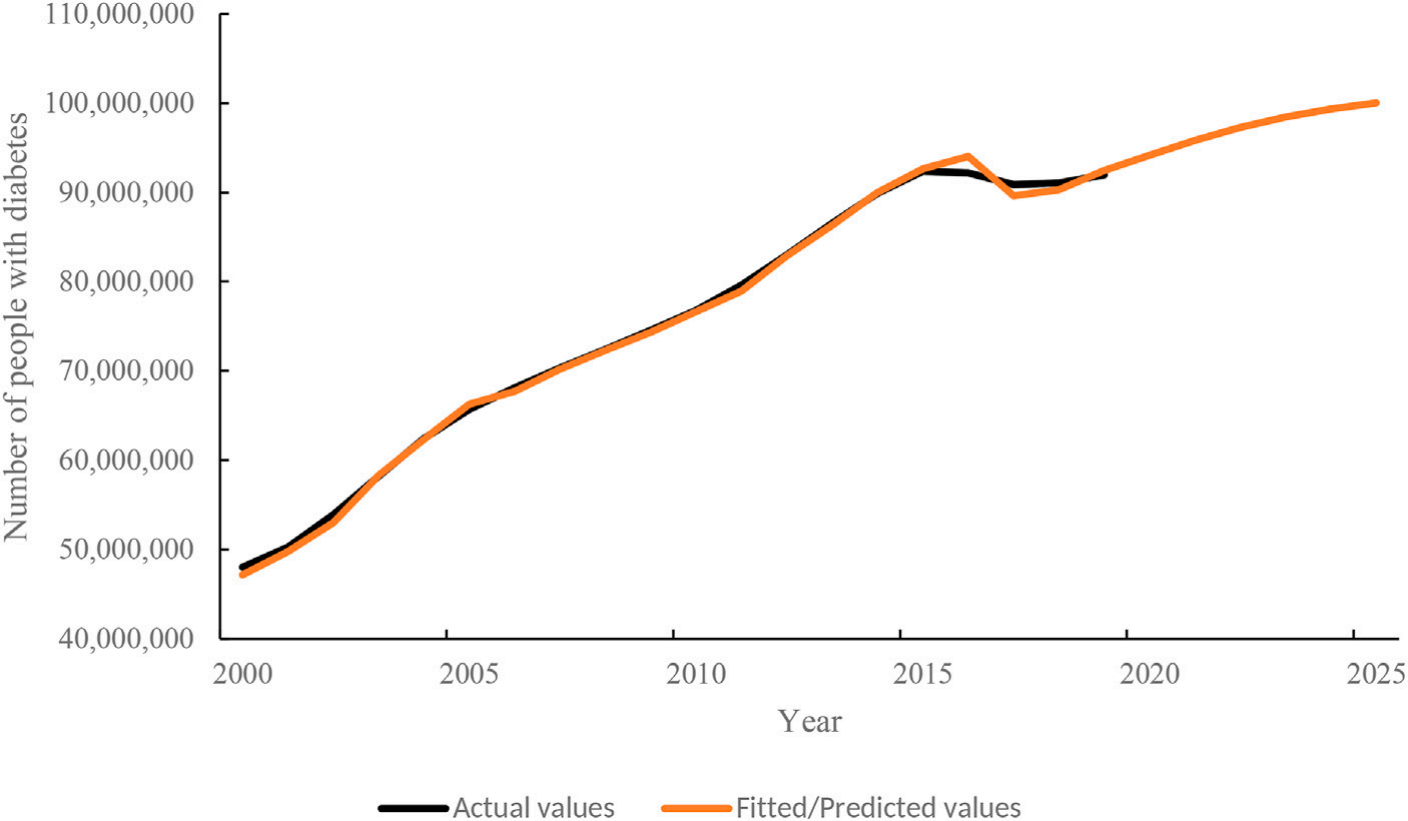
The prevalence trend of diabetes in the future, Predicting and Estimating Diabetes Burden, China, 2000–2025.

The general trend in the number of people with diabetes in China would increase from 2000 to 2025, as shown in Figure 3.

### Estimated Results of Economic Burden of Diabetes

The specific estimated results of the direct economic burden of diabetes in China were respectively 115,154,696,940, 117,917,067,172, 119,994,082,588, 121,789,554,504, 123,248,755,496, 124,388,575,044 and 125,254,880,168 from 2019 to 2025, as shown in Table 2. The estimated indirect economic burden of diabetes was about $41 billion, and the indirect economic burden per capita was about $447 in China in 2019, as shown in Table 3. The values of the total economic burden of diabetes in China were 156,302,288,326, 160,016,850,739, 162,835,420,381, 165,271,927,398, 167,252,105,102, 168,798,873,003 and 169,974,473,966 from 2019 to 2025, respectively (Supplemental Table S2).

**TABLE 2:** Direct economic burden of diabetes in China from 2019 to 2025, Predicting and Estimating Diabetes Burden, China, 2019–2025.

Year	Annual average direct economic burden (USD)	Number of patients	Direct economic burden (USD)
2019	1,252	91,976,595	**115,154,696,940**
2020	1,252	94,182,961	**117,917,067,172**
2021	1,252	95,841,919	**119,994,082,588**
2022	1,252	97,276,002	**121,789,554,504**
2023	1,252	98,441,498	**123,248,755,496**
2024	1,252	99,351,897	**124,388,575,044**
2025	1,252	100,043,834	**125,254,880,168**

The bold values in are the major results of the manuscript.

**TABLE 3:** Indirect economic burden of diabetes in China in 2019, Predicting and Estimating Diabetes Burden, China, 2019.

Age group (ages)	GNI per capita (USD)	DALYs (person-years)	Productivity weights	Indirect economic burden (USD)
0–14	10,410	15,217	0.15	23,761,346
15–44	10,410	1,265,391	0.75	9,879,540,233
45–59	10,410	3,056,314	0.80	25,452,982,992
≥60	10,410	5,563,215	0.10	5,791,306,815
Total	—	—	—	41,147,591,386

The bold values in are the major results of the manuscript.

## Discussion

The results show that the number of people with diabetes in China was on the rise from 2000 to 2019. The number of people with diabetes in China have surpassed 90 million per year since 2015. The possible reasons are as follows: Firstly, with the rapid societal transition, the rapid economic growth, and the rapid ageing of the population in China all lead to the increase in the number of people susceptible to diabetes [27, 28]. Secondly, dietary shifts towards westernized diets, such as increased intake of sugary drinks, dietary fats and red meat, and a shift towards recycled grains, all appear to increase the prevalence of diabetes in China [29]. Thirdly, sedentary lifestyle increases the risk of diabetes [30]. Fourthly, high-intensity social competition has led to a general increase in psychological stress in the population, especially in the middle-aged and elderly; high psychological stress can cause an increase in grain intake, which may be a risk factor for the development of diabetes [31]. Fifthly, the adult overweight and obesity rate increased rapidly in the last 20 years [32] and recent survey shows that more than 50% of adults are either overweight or obesity in China [33]. The onset of obesity and diabetes is associated with factors such as the c-Jun amino-terminal kinases (JNK) and the IκB kinase β (IKK-β), which cause insulin resistance and ultimately cause diabetes [34, 35]. Since the serious situation of diabetes in China, we should strengthen diabetes prevention and control, conduct early screening for diabetes, and prevent and manage diabetes complications actively.

In this study, ARIMA (2,1,1) model was used to predict the number of people with diabetes from 2020 to 2025. RMSE, MAE, MAPE, $R^{2}$ , average relative error of fit, and relative error of prediction indicated that the model fitted and predicted well. Therefore, the model can be used to predict the number of people with diabetes in China. In previous studies, the ARIMA model was commonly used to study the trend of infectious diseases such as the Corona Virus Disease 2019 (COVID-19) [36], tuberculosis [37], and malaria [38]. In this study, the raw data of diabetes can be transformed into a stationary time series after differencing, so we choose ARIMA model to fit and predict the prevalence trend of diabetes in China. Previous studies suggested ARIMA model could be used to predict the incidence of breast cancer and it performed well [39]. Therefore, we believe that the ARIMA model can also be used for fitted and predicted the trend of chronic disease prevalence.

According to our study, the total economic burden of diabetes in China are about $156 billion, $160 billion, $163 billion, $165 billion, $167 billion, $169 billion and $170 billion from 2019 to 2025 respectively, showing an increasing trend. The total economic burden of diabetes in 2019 accounted for about 1% of China’s Gross Domestic Product (GDP), of which the direct economic burden was about 12% of the total national health cost. The heavy economic burden of diabetes may be related to the following factors: Firstly, life expectancy per capita increased from 71.4 years in 2000 to 77.3 years in 2019, due to rapid aging in China, which is the most fundamental reason for the increasing trend of diabetes prevalence [40, 41]. Meanwhile, the treatment cost per capita and GNI per capita also rise. The results of this study are consistent with those of the International Diabetes Federation [5]. Secondly, diabetes as a chronic disease is characterized by a long course of disease, inevitably, the economic burden of diabetes increases every year. Thirdly, 76.40% of people with diabetes in China have reported at least one complication, and the treatment cost per capita for these patients is much higher than for patients with uncomplicated diabetes, thus creating a heavier economic burden [42].Based on the current serious situation of diabetes prevention and control in China, the study recommends the following measures. Firstly, authorities need to scientifically and actively promote the Notice of the General Office of the State Council on the Issuance of the Medium and Long-term Plan for the Prevention and Control of Chronic Diseases in China (2017–2025) [43]. Secondly, the primary health service system needs improvements, such as, to establish a database of diabetes monitoring system, and to encourage professionals to work in community health institutions through incentive mechanisms [44]. The authorities need to promote universal health coverage and raise awareness of diabetes prevention and control. Thirdly, the social security system should better combine health insurance and commercial insurance, and increase the reimbursement ratio of both, lower the starting standard of diabetes medical insurance, and set up a separate medical insurance funding mechanism for diabetes. Meanwhile, it is recommended that commercial insurance companies actively participate in the prevention of diabetes, provide health checkups for the insured, and lower the threshold of commercial insurance coverage, to reduce the economic burden of diabetes. Fourthly, we need to change the dietary structure and establish sensible dietary patterns to prevent and control diabetes [45]; encourage people to participate in sports to improved insulin sensitivity and optimized body mass index [46]; and reduce the risk of developing diabetes, so as to achieve the purpose of preventing and controlling diabetes.

### Limitations

There are some limitations in the study. Firstly, the ARIMA model is better at predicting data in the short term in the future [12], so we only predicted the number of people with diabetes from 2020 to 2025. Secondly, we used the treatment cost per capita and GNI per capita in 2019 to estimate the economic burden of diabetes from 2020 to 2025, without taking inflation into account. Thirdly, the proportion of people with a 2-week illness that is not treated is approximately 1.7% in China in 2018 [47]; therefore, using the number of people with diabetes instead of the number of inpatients with diabetes might overestimate the direct economic burden of diabetes, which requires further investigation of consultation and hospitalization rates to estimate the direct economic burden accurately.

### Conclusion

The ARIMA model prediction results indicated that the number of people with diabetes in China is would increase in the future. The ARIMA (2,1,1) model can be used to predict the number of people with diabetes in China. The economic burden of diabetes in China would also become heavier in the future. We call for government leadership and universal participation to improve diabetes-related health policies, and to achieve the purpose of preventing and controlling diabetes in China.
