# Supplementary material for: Predicting Diabetes and Estimating Its Economic Burden in China Using Autoregressive Integrated Moving Average Model
Source: Int J Public Health. 2022 Jan 20;66:1604449. doi: 10.3389/ijph.2021.1604449 (PMC8810486; doi:10.3389/ijph.2021.1604449)
Supplement: Supplementary file 1 [file DataSheet1.docx]

The data on the number of people with diabetes were shown in following Table S1.

The values of the total economic burden of diabetes in China from 2019 to 2025 were shown in following Table S2.

**Table S1** The data on the number of people with diabetes (Diabetes, China, 2000-2019)

| Year | Number of people  with diabetes |
| --- | --- |
|  |  |
| 2000 | 47990633 |
| 2001 | 50269421 |
| 2002 | 53901499 |
| 2003 | 58193128 |
| 2004 | 62428480 |
| 2005 | 65678126 |
| 2006 | 68103597 |
| 2007 | 70318751 |
| 2008 | 72414074 |
| 2009 | 74548519 |
| 2010 | 76781094 |
| 2011 | 79596503 |
| 2012 | 82982973 |
| 2013 | 86551169 |
| 2014 | 89875882 |
| 2015 | 92381953 |
| 2016 | 92191000 |
| 2017 | 90886584 |
| 2018 | 91078791 |
| 2019 | 91976595 |

**Table S2** Economic burden of diabetes in China from 2019 to 2025 (Diabetes, China, 2019-2025)

| Year | Direct economic burden (USD) | Indirect economic burden (USD) | Total economic burden (USD) |
| --- | --- | --- | --- |
| 2019 | 115,154,696,940 | 41,147,591,386 | **156,302,288,326** |
| 2020 | 117,917,067,172 | 42,099,783,567 | **160,016,850,739** |
| 2021 | 119,994,082,588 | 42,841,337,793 | **162,835,420,381** |
| 2022 | 121,789,554,504 | 43,482,372,894 | **165,271,927,398** |
| 2023 | 123,248,755,496 | 44,003,349,606 | **167,252,105,102** |
| 2024 | 124,388,575,044 | 44,410,297,959 | **168,798,873,003** |
| 2025 | 125,254,880,168 | 44,719,593,798 | **169,974,473,966** |
